# The influence of microbiota on the development and course of inflammatory diseases of periodontal tissues

**DOI:** 10.3389/froh.2023.1237448

**Published:** 2023-08-07

**Authors:** Andrii Demkovych, Dmytro Kalashnikov, Petro Hasiuk, Sergiy Zubchenko, Anna Vorobets

**Affiliations:** ^1^ Department of Orthopedic Dentistry, I. Horbachevsky Ternopil National Medical University, Ternopil, Ukraine; ^2^ Department of Propaedeutics of Prosthetic Dentistry, Poltava State Medical University, Poltava, Ukraine

**Keywords:** periodontium, generalized periodontitis, microbiota, inflammation, microorganisms

## Abstract

An important feature of the functioning of the organs and tissues of the oral cavity is the fact that all processes that take place in it are carried out in the constant presence of various microorganisms that cause the development of pathological processes in the body or are associated with them. In the pathogenesis of chronic generalized periodontitis, dental plaque penetrates the bottom of the gingival sulcus, penetrating under the epithelium into the stroma of the connective tissue, causing its inflammation. Bacteria produce a number of toxic substances that have a toxic effect on surrounding tissues. Most bacteria produce chain fatty acids that inhibit chemotaxis of leukocytes and phagocytes. Anaerobes and spirochetes secrete a number of substances (propionic acid and indole) that are extremely toxic to most tissues. Inflammation in the periodontal tissues is caused by the microbiota of the dental plaque biofilm. As periodontitis develops, an increase in the number of *P. gingivalis*, *P. intermedia* and *T. forsythia* was found in it, more than 100 times. Therefore, the given data prove that in the development and course of the inflammatory process in the periodontal tissues, complex dysbiotic and tissue-cellular interactions are involved, the dynamic balance of which depends on its outcome.

## Introduction

Inflammatory diseases of the periodontium are the initial stage of the destructive process, in particular generalized periodontitis, which leads to the loss of teeth and a violation of the communicative function of a person, and thus determines the social significance of the problem (1). An important feature of the functioning of the organs and tissues of the oral cavity is the fact that all processes that take place in it are carried out in the constant presence of various microorganisms that cause the development of pathological processes in the body or are associated with them (2, 3).

Based on the researches of the relationship between some types of microorganisms and destructive periodontal diseases (4, 5), two main points of view were formulated regarding the pathogenesis of inflammatory periodontal diseases: the first—there are certain bacterial pathogens that cause destructive damage to periodontal tissues; the second—the development of generalized periodontitis is caused by an imbalance of the body's protective and adaptive mechanisms (6). If we follow only the microbial etiology of periodontitis (7), the development of this disease requires a combination of the following conditions: the presence of periodontopathogenic bacteria in a quantity sufficient to initiate the inflammatory process; living conditions in the oral cavity should contribute to the growth and reproduction of pathogenic microbiota (8). Periodontal tissues must be free of microorganisms—antagonists of periodontopathogenic bacteria; microorganisms must be spatially localized so that they and (or) the products of their vital activity can act directly on target cells; the human body must be sensitive to microbes and their toxins. At the same time, it should be taken into account that the gingival barter has a number of features related to the structure of the mucous membrane of this component of the periodontium. The epithelium of the sulcular part of the gums, located around the neck of the tooth, does not have keratinized cells. The distance between the epithelial cells of this department is greater than in other departments of the gums mucous membrane. These factors determine the higher permeability of the epithelium for microbial toxins and leukocytes (9).

Plaque microorganisms, as a result of the active release of various enzymes that contribute to the development of periodontal microcirculatory disorders, trigger a number of inflammatory reactions, cause depolymerization of glycosaminoglycans, periodontal tissue proteins, primarily collagen. This mechanism of the development of the pathological process occupies an important place in the pathogenesis of the development of periodontal diseases of dystrophic-inflammatory origin (10, 11).

### Evaluation of the impact of plaque microorganisms on the pathogenesis of periodontal tissue deseases

The main pathogenetic link, the facet of the transformation of the protective plaque biofilm formed by the indigenous microbiota of the oral cavity, is the overcoming by representatives of the microbiota of the epithelial cover and the spread of the inflammatory infiltrate in the connective tissue of the periodontium behind the tooth-gingival junction of the gingival sulcus (1, 4, 12).

Ornithine decarboxylase is a key enzyme in the synthesis of regulatory polyamines, such as putrescine, spermine, spermidine, and others, which regulate replication and transcription processes and, as a result, cell proliferation. There are also data on the role of polyamines associated with ornithine decarboxylase in the mechanism of action of epidermal growth factor. In *in vitro* experiments, polyamines stimulate the activity of DNA-dependent RNA polymerase. The essential role of polyamines is the initiation of peptide synthesis by changing the conformation of ribosomes. Thus, polyamines play an important regulatory role in processes related to the biosynthesis of proteins and nucleic acids (1).

Bacteria produce a number of toxic substances that have a toxic effect on surrounding tissues. Most bacteria produce chain fatty acids that inhibit chemotaxis of leukocytes and phagocytes. Anaerobes and spirochetes secrete a number of substances (propionic acid and indole) that are extremely toxic to most tissues (13).

As a result of tissue damage, thrombin, kinins (inflammation mediators), and activated complement fractions are released. These proteins, together with the products of bacterial life, play the role of chemotactic factors for polynuclear cells, macrophages and other cellular elements involved in the development of the inflammatory process (13, 14).

The oral cavity can be considered as a complex ecological system in which external factors (biological, individual, social) interact with internal factors (periodontal, dentin metabolites, bacteria, local immune system of the mucous membrane, oral cavity epithelium, saliva, nerve endings). As in the environment, all components of the system are in dynamic equilibrium. Components of this system are not only bacteria, but also any pathogens, including viruses and fungi, and a stable microbial environment in the oral cavity is created by many pathogenic agents (6, 15).

More than 1,000 types of groups of microorganisms associated with the mucous membrane epithelium or located on the tooth surface have been found in the oral cavity. 417 species of bacteria were isolated from tartar (2, 10, 16). Individual differences in the number of microorganisms in the oral cavity of healthy adults with intact teeth depend on many factors: the nature of the diet, the intervals between meals, the width of the interdental spaces, and the hygienic care of the oral cavity (3, 17).

Meanwhile, the number and species composition of the microbiota of the oral cavity of each healthy person are relatively stable, since there are a number of factors that ensure the stability of the composition of the oral microbiota. One of the main roles in maintaining the stability of the microbial composition of the oral cavity is played by the inherent antagonism of the permanent microbiota against pathogenic and opportunistic microbes (3).

The permanent microbiota of the oral cavity includes representatives of several groups of microorganisms: (1) bacteria; (2) fungi; (3) protozoa; (4) viruses.

According to a number of authors (2, 16), about half of the representatives of the resident (normal) flora are facultative and obligate-anaerobic streptococci, which include *S. salivarius*, *S. mutans*, *S. mitis*, *S. sanguis* and *peptostreptococci*. The other half of the resident microbiota consists of *veillonella* (about 25%) and *diphtheroids* (about 25%). Obligate anaerobes in the oral cavity are also constantly represented by the bacteroides group. *Lactobacilli, staphylococci, fusobacteria, bacteroids*, yeasts, fungi and protozoa belong to secondary representatives of resistant microbiota (18).

One of the important functions of normal microbiota is maintaining the “working” state of specific and non-specific, humoral and cellular mechanisms of immunity. *Bifidobacteria* stimulate the lymphoid apparatus, the synthesis of immunoglobulins, increase the level of properdin and complement, increase the activity of lysozyme and help reduce the permeability of vascular-tissue barriers to toxic products of pathogenic and opportunistic microorganisms, which prevents the development of bacteremia and sepsis (3, 19).

A sharp general increase in the number of microorganisms occurs in the presence of abnormalities and defects in the oral cavity, which contribute to the retention of food residues and make it difficult to wash out microorganisms with saliva. This is observed in congenital defects of the maxillofacial region, multiple carious lesions, formation of periodontal pockets, poor-quality prosthetics, as well as under the influence of general pathology: when the body's reactivity changes, endocrine diseases, etc (20, 21).

It is possible that one of the most important mechanisms is a violation of the formation of a biofilm, which is a microbiological population associated with an organic and inorganic substrate. These microcolonies have their own microenvironments, differing in pH levels, digestibility of nutrients, and oxygen concentrations. Bacteria in the biofilm exchange genetic material, “communicate with each other» are using chemical stimuli (signals). These chemical irritations cause bacteria to produce potentially harmful proteins and enzymes (22, 23).

In addition, there is a complex multi-level system of interaction between bacteria and epithelial cells (24), which includes cytokines, ligands of apoptosis receptors, bacterial metabolites of bacteria, as well as special receptors—TLRs, which determine the invasion of bacteria into cells (25).

### Interconnection between inflammatory diseases of periodontal tissues and the amount and nature of the microbial composition dental plaque

There are two main theories that assess the relationship between inflammatory periodontal diseases and the amount and nature of the microbial composition of dental plaque in different ways (3).

The theory of a non-specific microbial composition was proposed by W. Loesche in 1976. The author suggests that periodontal recovery depends on “the amount of harmful substances produced by bacteria”. This means that as long as the amount of these agents does not exceed the protective capacity of saliva and tissues, the periodontium remains in a normal state. According to this concept, the condition of the periodontium depends on the level of oral hygiene. In most clinical cases, this theory is unquestionably confirmed, and it is on its basis that a general scheme of treatment and preventive measures is built: removal of dental deposits and the use of antibacterial agents (26).

The theory of the specific microbial composition of the plaque is that only a specific plaque composition is pathogenic, and its pathogenicity is associated with the presence or increase in the plaque composition of only certain microorganisms. The author of this theory is also W. Loesche, he proclaimed it on the basis of methods of isolating specific microorganisms in the composition of dental plaque (27).

This theory received its main development with the appearance of evidence of the role of *Aggregatibacter actinomycetemcomitans* in the pathogenesis of juvenile periodontitis, and a little later—about the similar role of *Porphyromonas gingivalis* in typical forms. Predominance of *A. actinomycetemcomitans* in tissues is a bad prognostic sign even in typical forms of generalized periodontitis. It is believed that the development and progression of periodontal diseases can be associated with the influence of 6–10 microorganisms that manifest their pathogenic effect in any combination. Later, this theory gained the most popularity (3).

It was established that *Porphyromonas gingivalis, Aggregatibacter actinomycetemcomitans, Prevotella intermedia, Tannerella forsythia, Eikenella corrodens, Fusobacterium nucleatum* are most often found in places of the greatest periodontal destruction. However, the same bacteria are also present in healthy people in intact periodontium, because there is a balance between macro- and microorganism. Without clear evidence of the etiotropic nature of a specific microorganism to a certain form of periodontal disease, we can only talk about the “main” microbial pathogens in certain clinical manifestations of the disease. Inflammation in the periodontal tissues is caused by the microbiota of the dental plaque biofilm (28). As periodontitis develops, an increase in the number of *P. gingivalis, P. intermedia and T. forsythia* was found in it, more than 100 times (3, 29, 30).

*Aggregatibacter actinomycetemcomitans (A. a.)* are immobile gram-negative facultatively anaerobic bacilli that play a key role in the development of localized aggressive generalized periodontitis. The phenotypic variability of strains of *A. a.* may affect the pathogenesis of periodontitis. 6 serotypes of *A. a.* is also found in patients who do not suffer from periodontitis. Early clinical studies established the ability of *A. a.* to penetrate the gingival epithelium, and in a very unusual way, with specific intracellular localization (31, 32). During this dynamic process, *A. a.* attaches itself to the body cell. First, the microvilli of the epitheliocyte are smoothed, then the bacterium is surrounded by protrusions of the membrane and penetrates inside the cell, leading to the formation of a vacuole in it. After that, the vacuole quickly collapses, and the bacteria enter the cytoplasm (33). Next, the restructuring of the functions of the host cell, typical for intracellular parasites, takes place. In the last few decades, about 200 infectious diseases of the extraoral cavity associated with *A. a.* have been identified. The results of *in vitro* studies allowed us to assume that as a result of penetration into epithelial cells and the development of the process from the inside and intercellular spread of *A. a.* in the connective tissue of the gums, the destruction characteristic of periodontal diseases develops.

*Porphyromonas gingivalis* are immobile gram-negative anaerobic rods. The surface of *P. gingivalis* is covered peritrichially with fimbriae. They are the most frequent, after *A. a.*, causative agents of chronic generalized periodontitis. Especially many of them can be found in fresh lesions. Of all pathogens, they are most closely associated with chronic periodontitis (34, 35). Intracellularly located *Porphyromonas gingivalis* are able to control cell metabolism, which is directly related to the development of the disease (36, 37). Thus, after the invasion of *Porphyromonas gingivalis* in the gingival epitheliocytes, the secretion of interleukin-8 is suppressed, this in general weakens the natural protection of the periodontium. In the conditions created by the macroorganism, the signal about the presence of bacteria is removed and leukocytes are not directed to destroy them. *P. gingivalis* can prevent the migration of polymorphonuclear leukocytes through the epithelial barrier (38). Detection of *P. gingivalis* indicates the risk of progression of chronic generalized periodontitis. Their number increases significantly with periodontal diseases, especially in fresh lesions. It has been shown that proteolytic enzymes can destroy various proteins of the body and possibly disrupt the functions of its cells. *P. gingivalis* synthesizes proteases that destroy immunoglobulins, gingipains that induce the production of interleukin-6 by neutrophils, hemolysins, and endotoxins (39, 40).

*Tannerella forsythia (T. f.)* is a spindle-shaped, immobile gram-negative bacterium, an obligate anaerobe. The surface S-layer of this microorganism promotes aggregation and invasion of epithelial cells, promotes erythrocyte agglutination (41). When co-cultivated with macrophages and epithelial cells *T. f.* causes the release of anti-inflammatory cytokines, chemokines, prostaglandins E. It takes about 12 days to cultivate a small colony of bacteria. Studies have shown that this periodontopathogen is found in patients with periodontitis that did not respond to treatment (42).

*Prevotella intermedia* is a gram-negative bacterium. It is an obligate anaerobe. Numerous studies have shown that this periodontopathogen is resistant to antibiotics. *Prevotella intermedia* can penetrate the epithelial cells of oral cavity tissues (43). The presence of *Prevotella intermedia* in the body contributes to increased secretion of matrix metalloproteinase-8 and matrix metalloproteinase-9 in periodontal tissues and blood plasma. In vitro studies revealed *P. intermedia* in patients with periodontitis in the intercellular space of periodontal tissues and in blood serum.

*Treponema denticola* is a gram-negative bacterium, an obligate anaerobe. *Treponema denticola* promotes the production of metalloproteinases by polymorphonuclear leukocytes, causing the destruction of the intercellular substance of the connective tissue. *T. denticola* can agglutinate and lyse red blood cells. The oral cavity spirochete *Treponema denticola* does not penetrate living epitheliocytes, but induces the depolymerization of actin microfilaments, along with the weakening of the attachment of the epitheliocytes themselves. The surface protein of *T. denticola* can move into the epitheliocyte membrane with its subsequent depolarization and formation of ion channels. As a result of such an attack, the functions of epitheliocytes are disturbed, and in case of chronic generalized periodontitis, many treponems are found in the material (43, 44). The ability of this periodontopathogen to activate macrophages, which, in turn, secrete substances that contribute to the breakdown of collagen (nitric oxide and cytokines) has been proven. *T. denticola* against the background of impaired and normal function of neutrophils causes deep lesions (45, 46, 47). *Treponema denticola* can attach to the endothelium, connect with cells along their entire length. It forms aggregates with *P. gingivalis* and *Fusobacterium* spp., which can be important for the formation of dental plaque, as well as for the nutrition of bacteria.

*Chlamydia trachomatis* is an obligate intracellular parasite measuring 250–300 nm. It is a non-moving gram-negative form, which during primary infection affects the cells of the main barrier systems of the body. Because it has RNA, DNA, a cell wall, and ribosomes similar to those of gram-negative bacteria, *C. trachomatis* is classified as a bacterium. *C. trachomatis* can exist in the body for a long time in a hidden form. Under unfavorable conditions (exposure to antibiotics, overheating, hypothermia) *C. trachomatis* can transform into so-called L-forms. This phenomenon promotes long-term intracellular parasitism without conflicts with the host's immune system. When body cells divide, inactive *C. trachomatis* is transferred to daughter cells. Active reproduction and the so-called reversion of *C. trachomatis* from L-forms are possible only under conditions of immunosuppression. The invasiveness of *C. trachomatis* is associated with the structure of the carbohydrate part of the main lipopolysaccharide of their outer membrane (43, 48).

*C. trachomatis* has the possibility of intracellular parasitism. Combining with viruses, it cannot synthesize its ATP, depends on the energy resources of the cell and in the process of parasitism completely destroys it. The presence of a cell membrane unites *C. trachomatis* with bacteria. This allows the use of antibiotics to treat chlamydiosis. *C. trachomatis* has a pronounced tropism to the epithelium of certain organs. The main morphological forms of *C. trachomatis* are elementary bodies and reticular bodies. Elementary bodies of *C. trachomatis* have all infectious qualities. They are spherical structures with a diameter of 250–300 nm, covered on the outside with a three-layer membrane, 8 nm thick (49, 50, 51).

In the process of invasion, bacteria produce compounds that reduce or completely block the activity of the body's defense systems (52). If the saprophytic representatives of the microbiota secrete an exotoxin to which periodontal tissues are tolerant, then a feature of periodontopathogenic microorganisms is the secretion of endotoxin, which actively damages cells, connective tissue formations, and the main substance (11, 53, 54).

Many microorganisms present in large quantities in periodontal diseases destroy immunoglobulins with their enzymes. The most active are microbial proteases, which reduce the production of IgA and IgG, thereby reducing the barrier function of the mucous membrane of the oral cavity and facilitating the penetration of toxic products, lytic enzymes, and subgingival microbiota into the tissues (55, 56).

## Conclusions

Therefore, the given data prove that in the development and course of the inflammatory process in the periodontal tissues, complex dysbiotic and tissue-cellular interactions are involved, the dynamic balance of which depends on its outcome.
